# (*S*_P_)-Menthyl benzyl­(phenyl)­phospho­nate

**DOI:** 10.1107/S1600536810009372

**Published:** 2010-03-17

**Authors:** Wei-Min Wang, Chang-Qiu Zhao

**Affiliations:** a College of Chemistry and Chemical Engineering, Liaocheng University, Shandong 252059, People’s Republic of China

## Abstract

The title compound, C_23_H_31_O_2_P, has three fully extended substituents around the P-atom chiral centre, forming an *S*_P_ configuration. The phenyl rings are inclined at a dihedral angle of 3.2 (3)°.

## Related literature

For general background to phospho­rus-sulfur compounds, see: Dilworth & Wheatley (2000 ▶); Chae *et al.* (1994 ▶). For alkyl­ates of phospho­rus-sulfur compounds, see: Aitken (2005 ▶).
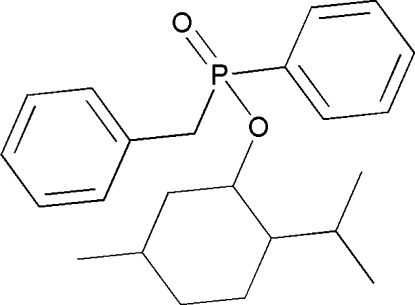


## Experimental

### 

#### Crystal data

C_23_H_31_O_2_P
*M* *_r_* = 370.45
Monoclinic, 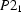

*a* = 12.4777 (11) Å
*b* = 5.7970 (7) Å
*c* = 15.4190 (19) Å
β = 100.727 (1)°
*V* = 1095.8 (2) Å^3^
*Z* = 2
Mo *K*α radiation
μ = 0.14 mm^−1^
*T* = 298 K
0.43 × 0.11 × 0.10 mm

#### Data collection

Siemens SMART CCD area-detector diffractometer
Absorption correction: multi-scan (*SADABS*; Sheldrick, 1996 ▶) *T*_min_ = 0.943, *T*_max_ = 0.986
5541 measured reflections
3576 independent reflections
2596 reflections with *I* > 2σ(*I*)
*R*_int_ = 0.085

#### Refinement

*R*[*F* ^2^ > 2σ(*F* ^2^)] = 0.066
*wR*(*F* ^2^) = 0.170
*S* = 0.89
3576 reflections
238 parameters
1 restraint
H-atom parameters constrained
Δρ_max_ = 0.28 e Å^−3^
Δρ_min_ = −0.23 e Å^−3^
Absolute structure: Flack (1983 ▶), 1432 Friedel pairs
Flack parameter: −0.17 (16)

### 

Data collection: *SMART* (Siemens, 1996 ▶); cell refinement: *SAINT* (Siemens, 1996 ▶); data reduction: *SAINT*; program(s) used to solve structure: *SHELXS97* (Sheldrick, 2008 ▶); program(s) used to refine structure: *SHELXL97* (Sheldrick, 2008 ▶); molecular graphics: *SHELXTL* (Sheldrick, 2008 ▶); software used to prepare material for publication: *SHELXTL*.

## Supplementary Material

Crystal structure: contains datablocks I, global. DOI: 10.1107/S1600536810009372/bx2269sup1.cif

Structure factors: contains datablocks I. DOI: 10.1107/S1600536810009372/bx2269Isup2.hkl

Additional supplementary materials:  crystallographic information; 3D view; checkCIF report

## Figures and Tables

**Fig. 1.**
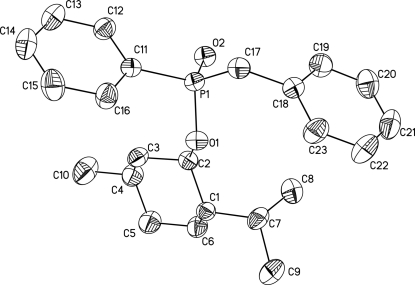
The molecular structure of the compound. H atoms have been omitted for clarity.

## supplementary crystallographic information

### Comment

Phosphorus-sulfur compounds, especially P-chiral, have extensive applications (Dilworth *et al.* 2000; Chae *et al.* 1994), wherein their alkylates are of significant senes, for example, they usually act as common ligands (Aitken *et al.* 2005). The title compound is a P-chiral compounds, which can be synthesized by (*R*_p_)-O-menthyl *S*-ethyl phenylphosphonothioate and benzylmagnesium chloride. The compound is comprised of fully extended substituents: phenyl, menthyloxy and benzyl, and O atom which form a irregular tetrahedron, (Table 1). The phenyl rings makes a dihedral angle of 3.2 (3)°.The six-membered menthyloxy ring is in a chair conformation. The molecular structure is stabilized by a intramolecular C—H··· O hydrogen bond [C···O =2.893 (5) Å , C—H··· O =104°]. There are no further significant intermolecular interactions.

### Experimental

(*R*_p_)-O-menthyl *S*-ethyl phenylphosphonothioate (0.3 mmol) was added to a stirred ether solution of benzylmagnesium chloride (0.6 mmol) in a Schlenk tube under nitrogen and the mixture was stirred for 24 h at room temperature. After washing with water, the resulting solution was purified by silica gel plate to afford optically pure product. The crystal suitable  for X-ray diffraction was obtained from recrystallization with ethyl ether/hexane.

### Refinement

All H atoms attached to C atoms were fixed geometrically and treated as riding with C—H = 0.93-0.98 Å, with *U*_iso_(H) = 1.5 *U*_eq_(methyl) and *U*_iso_(H) = 1.2 *U*_eq_(C) for all other H atoms.

### Crystal data

**Table:**

C_23_H_31_O_2_P	*F*(000) = 400
*M**_r_* = 370.45	*D*_x_ = 1.123 Mg m^−^^3^
Monoclinic, *P*2_1_	Mo *K*α radiation, λ = 0.71073 Å
Hall symbol: P 2yb	Cell parameters from 1629 reflections
*a* = 12.4777 (11) Å	θ = 2.7–25.0°
*b* = 5.7970 (7) Å	µ = 0.14 mm^−^^1^
*c* = 15.4190 (19) Å	*T* = 298 K
β = 100.727 (1)°	Block, colorless
*V* = 1095.8 (2) Å^3^	0.43 × 0.11 × 0.10 mm
*Z* = 2	

### Data collection

**Table:**

Siemens SMART CCD area-detector diffractometer	3576 independent reflections
Radiation source: fine-focus sealed tube	2596 reflections with *I* > 2σ(*I*)
graphite	*R*_int_ = 0.085
phi and ω scans	θ_max_ = 25.0°, θ_min_ = 1.3°
Absorption correction: multi-scan (*SADABS*; Sheldrick, 1996)	*h* = −14→14
*T*_min_ = 0.943, *T*_max_ = 0.986	*k* = −6→6
5541 measured reflections	*l* = −15→18

### Refinement

**Table:**

Refinement on *F*^2^	Secondary atom site location: difference Fourier map
Least-squares matrix: full	Hydrogen site location: inferred from neighbouring sites
*R*[*F*^2^ > 2σ(*F*^2^)] = 0.066	H-atom parameters constrained
*wR*(*F*^2^) = 0.170	*w* = 1/[σ^2^(*F*_o_^2^) + (0.1052*P*)^2^] where *P* = (*F*_o_^2^ + 2*F*_c_^2^)/3
*S* = 0.89	(Δ/σ)_max_ < 0.001
3576 reflections	Δρ_max_ = 0.28 e Å^−^^3^
238 parameters	Δρ_min_ = −0.23 e Å^−^^3^
1 restraint	Absolute structure: Flack (1983), 1432 Friedel pairs
Primary atom site location: structure-invariant direct methods	Flack parameter: −0.17 (16)

### Fractional atomic coordinates and isotropic or equivalent isotropic displacement parameters (Å^2^)

**Table:**

	*x*	*y*	*z*	*U*_iso_*/*U*_eq_	
P1	0.14134 (8)	0.32764 (18)	0.12780 (6)	0.0440 (3)	
O1	0.2011 (2)	0.2676 (5)	0.22583 (18)	0.0495 (7)	
O2	0.1092 (2)	0.5709 (5)	0.11455 (18)	0.0539 (8)	
C1	0.2811 (3)	0.3635 (8)	0.3776 (3)	0.0513 (11)	
H1	0.3316	0.2327	0.3810	0.062*	
C2	0.2606 (3)	0.4484 (9)	0.2817 (3)	0.0494 (10)	
H2	0.2140	0.5856	0.2776	0.059*	
C3	0.3653 (4)	0.5136 (9)	0.2527 (3)	0.0604 (13)	
H3A	0.3492	0.5651	0.1918	0.072*	
H3B	0.4119	0.3788	0.2556	0.072*	
C4	0.4259 (4)	0.7041 (10)	0.3099 (3)	0.0736 (15)	
H4	0.3776	0.8387	0.3045	0.088*	
C5	0.4456 (4)	0.6286 (13)	0.4061 (3)	0.0831 (17)	
H5A	0.4964	0.5004	0.4142	0.100*	
H5B	0.4786	0.7548	0.4430	0.100*	
C6	0.3419 (4)	0.5571 (10)	0.4352 (3)	0.0694 (14)	
H6A	0.2942	0.6901	0.4329	0.083*	
H6B	0.3590	0.5049	0.4959	0.083*	
C7	0.1778 (4)	0.2749 (10)	0.4090 (3)	0.0665 (14)	
H7	0.1455	0.1554	0.3672	0.080*	
C8	0.0910 (5)	0.4614 (15)	0.4086 (4)	0.104 (2)	
H8A	0.0268	0.3938	0.4239	0.157*	
H8B	0.0731	0.5286	0.3508	0.157*	
H8C	0.1187	0.5787	0.4508	0.157*	
C9	0.2060 (5)	0.1607 (12)	0.4997 (3)	0.0865 (18)	
H9A	0.2317	0.2756	0.5435	0.130*	
H9B	0.2620	0.0472	0.4992	0.130*	
H9C	0.1422	0.0876	0.5134	0.130*	
C10	0.5316 (5)	0.7791 (15)	0.2790 (4)	0.113 (3)	
H10A	0.5812	0.6511	0.2838	0.170*	
H10B	0.5649	0.9037	0.3153	0.170*	
H10C	0.5143	0.8289	0.2186	0.170*	
C11	0.2347 (3)	0.2350 (7)	0.0575 (3)	0.0445 (10)	
C12	0.2528 (4)	0.3793 (9)	−0.0106 (3)	0.0615 (14)	
H12	0.2167	0.5200	−0.0204	0.074*	
C13	0.3260 (4)	0.3096 (13)	−0.0641 (3)	0.0790 (15)	
H13	0.3387	0.4050	−0.1097	0.095*	
C14	0.3790 (4)	0.1029 (11)	−0.0502 (4)	0.0762 (15)	
H14	0.4277	0.0581	−0.0859	0.091*	
C15	0.3601 (4)	−0.0387 (11)	0.0170 (4)	0.0776 (15)	
H15	0.3964	−0.1791	0.0267	0.093*	
C16	0.2876 (4)	0.0258 (9)	0.0701 (3)	0.0652 (13)	
H16	0.2744	−0.0724	0.1146	0.078*	
C17	0.0306 (3)	0.1248 (8)	0.1089 (3)	0.0511 (10)	
H17A	−0.0110	0.1479	0.0498	0.061*	
H17B	0.0603	−0.0303	0.1117	0.061*	
C18	−0.0457 (3)	0.1452 (8)	0.1747 (3)	0.0490 (10)	
C19	−0.1144 (4)	0.3305 (10)	0.1742 (3)	0.0668 (12)	
H19	−0.1153	0.4478	0.1329	0.080*	
C20	−0.1828 (4)	0.3417 (13)	0.2360 (4)	0.0828 (16)	
H20	−0.2284	0.4684	0.2363	0.099*	
C21	−0.1837 (5)	0.1675 (13)	0.2966 (4)	0.0861 (18)	
H21	−0.2302	0.1752	0.3371	0.103*	
C22	−0.1159 (5)	−0.0153 (12)	0.2966 (4)	0.0910 (19)	
H22	−0.1155	−0.1324	0.3379	0.109*	
C23	−0.0479 (4)	−0.0298 (10)	0.2365 (3)	0.0689 (13)	
H23	−0.0028	−0.1576	0.2369	0.083*	

### Atomic displacement parameters (Å^2^)

**Table:**

	*U*^11^	*U*^22^	*U*^33^	*U*^12^	*U*^13^	*U*^23^
P1	0.0524 (5)	0.0365 (6)	0.0430 (5)	0.0020 (6)	0.0084 (4)	−0.0002 (5)
O1	0.0601 (16)	0.0395 (19)	0.0464 (15)	0.0000 (13)	0.0034 (12)	−0.0034 (12)
O2	0.0638 (16)	0.0401 (18)	0.0577 (18)	0.0061 (15)	0.0106 (14)	−0.0018 (13)
C1	0.054 (2)	0.055 (3)	0.044 (2)	0.005 (2)	0.0090 (17)	−0.002 (2)
C2	0.052 (2)	0.048 (3)	0.047 (2)	0.003 (2)	0.0061 (19)	−0.008 (2)
C3	0.067 (3)	0.068 (4)	0.047 (2)	−0.007 (2)	0.012 (2)	0.000 (2)
C4	0.069 (3)	0.087 (4)	0.064 (3)	−0.018 (3)	0.009 (2)	−0.013 (3)
C5	0.074 (3)	0.114 (5)	0.058 (3)	−0.023 (3)	0.004 (2)	−0.015 (3)
C6	0.066 (3)	0.090 (4)	0.052 (3)	−0.009 (3)	0.011 (2)	−0.014 (3)
C7	0.078 (3)	0.078 (4)	0.047 (2)	−0.008 (3)	0.022 (2)	−0.005 (2)
C8	0.075 (3)	0.136 (6)	0.107 (5)	0.023 (4)	0.031 (3)	0.026 (4)
C9	0.111 (4)	0.089 (5)	0.067 (4)	−0.004 (4)	0.036 (3)	0.001 (3)
C10	0.101 (4)	0.152 (8)	0.089 (4)	−0.061 (5)	0.022 (3)	−0.019 (5)
C11	0.047 (2)	0.042 (3)	0.044 (2)	−0.0032 (18)	0.0047 (17)	0.0013 (17)
C12	0.067 (3)	0.062 (4)	0.058 (3)	0.002 (2)	0.017 (2)	0.006 (2)
C13	0.088 (3)	0.091 (4)	0.066 (3)	−0.005 (4)	0.034 (3)	0.006 (4)
C14	0.070 (3)	0.080 (4)	0.087 (4)	−0.008 (3)	0.037 (3)	−0.020 (3)
C15	0.075 (3)	0.058 (3)	0.107 (4)	0.010 (3)	0.032 (3)	−0.007 (3)
C16	0.070 (3)	0.056 (3)	0.075 (3)	0.008 (3)	0.029 (3)	0.008 (2)
C17	0.057 (2)	0.046 (3)	0.049 (2)	0.004 (2)	0.0082 (19)	−0.0035 (19)
C18	0.055 (2)	0.043 (3)	0.048 (2)	−0.005 (2)	0.0085 (18)	−0.0033 (19)
C19	0.070 (3)	0.056 (3)	0.076 (3)	0.010 (3)	0.017 (2)	0.009 (3)
C20	0.069 (3)	0.084 (4)	0.100 (4)	0.003 (3)	0.028 (3)	−0.013 (4)
C21	0.078 (4)	0.103 (5)	0.088 (4)	−0.013 (4)	0.041 (3)	−0.007 (4)
C22	0.112 (5)	0.088 (5)	0.082 (4)	−0.012 (4)	0.042 (3)	0.018 (4)
C23	0.085 (3)	0.053 (3)	0.072 (3)	−0.002 (3)	0.025 (3)	0.013 (3)

### Geometric parameters (Å, °)

**Table:**

P1—O2	1.470 (3)	C9—H9C	0.9600
P1—O1	1.595 (3)	C10—H10A	0.9600
P1—C17	1.796 (4)	C10—H10B	0.9600
P1—C11	1.814 (4)	C10—H10C	0.9600
O1—C2	1.467 (5)	C11—C16	1.377 (6)
C1—C2	1.533 (5)	C11—C12	1.394 (6)
C1—C6	1.540 (6)	C12—C13	1.400 (7)
C1—C7	1.548 (6)	C12—H12	0.9300
C1—H1	0.9800	C13—C14	1.366 (8)
C2—C3	1.507 (6)	C13—H13	0.9300
C2—H2	0.9800	C14—C15	1.377 (8)
C3—C4	1.524 (7)	C14—H14	0.9300
C3—H3A	0.9700	C15—C16	1.380 (7)
C3—H3B	0.9700	C15—H15	0.9300
C4—C5	1.522 (7)	C16—H16	0.9300
C4—C10	1.546 (7)	C17—C18	1.519 (6)
C4—H4	0.9800	C17—H17A	0.9700
C5—C6	1.504 (7)	C17—H17B	0.9700
C5—H5A	0.9700	C18—C19	1.374 (6)
C5—H5B	0.9700	C18—C23	1.396 (6)
C6—H6A	0.9700	C19—C20	1.395 (7)
C6—H6B	0.9700	C19—H19	0.9300
C7—C8	1.529 (8)	C20—C21	1.377 (9)
C7—C9	1.528 (7)	C20—H20	0.9300
C7—H7	0.9800	C21—C22	1.356 (9)
C8—H8A	0.9600	C21—H21	0.9300
C8—H8B	0.9600	C22—C23	1.370 (7)
C8—H8C	0.9600	C22—H22	0.9300
C9—H9A	0.9600	C23—H23	0.9300
C9—H9B	0.9600		
			
O2—P1—O1	114.13 (15)	C7—C9—H9A	109.5
O2—P1—C17	115.1 (2)	C7—C9—H9B	109.5
O1—P1—C17	102.76 (18)	H9A—C9—H9B	109.5
O2—P1—C11	112.96 (18)	C7—C9—H9C	109.5
O1—P1—C11	105.23 (17)	H9A—C9—H9C	109.5
C17—P1—C11	105.6 (2)	H9B—C9—H9C	109.5
C2—O1—P1	119.8 (3)	C4—C10—H10A	109.5
C2—C1—C6	107.3 (4)	C4—C10—H10B	109.5
C2—C1—C7	114.1 (3)	H10A—C10—H10B	109.5
C6—C1—C7	114.2 (4)	C4—C10—H10C	109.5
C2—C1—H1	106.9	H10A—C10—H10C	109.5
C6—C1—H1	106.9	H10B—C10—H10C	109.5
C7—C1—H1	106.9	C16—C11—C12	119.6 (4)
O1—C2—C3	112.1 (3)	C16—C11—P1	121.4 (3)
O1—C2—C1	108.2 (4)	C12—C11—P1	119.0 (3)
C3—C2—C1	111.6 (3)	C11—C12—C13	119.1 (5)
O1—C2—H2	108.3	C11—C12—H12	120.5
C3—C2—H2	108.3	C13—C12—H12	120.5
C1—C2—H2	108.3	C14—C13—C12	120.7 (5)
C2—C3—C4	111.9 (4)	C14—C13—H13	119.7
C2—C3—H3A	109.2	C12—C13—H13	119.7
C4—C3—H3A	109.2	C13—C14—C15	119.7 (5)
C2—C3—H3B	109.2	C13—C14—H14	120.2
C4—C3—H3B	109.2	C15—C14—H14	120.2
H3A—C3—H3B	107.9	C14—C15—C16	120.5 (6)
C5—C4—C3	109.2 (5)	C14—C15—H15	119.7
C5—C4—C10	112.9 (4)	C16—C15—H15	119.7
C3—C4—C10	112.6 (5)	C11—C16—C15	120.4 (5)
C5—C4—H4	107.3	C11—C16—H16	119.8
C3—C4—H4	107.3	C15—C16—H16	119.8
C10—C4—H4	107.3	C18—C17—P1	113.5 (3)
C6—C5—C4	112.2 (4)	C18—C17—H17A	108.9
C6—C5—H5A	109.2	P1—C17—H17A	108.9
C4—C5—H5A	109.2	C18—C17—H17B	108.9
C6—C5—H5B	109.2	P1—C17—H17B	108.9
C4—C5—H5B	109.2	H17A—C17—H17B	107.7
H5A—C5—H5B	107.9	C19—C18—C23	118.7 (4)
C5—C6—C1	112.8 (4)	C19—C18—C17	121.7 (4)
C5—C6—H6A	109.0	C23—C18—C17	119.6 (4)
C1—C6—H6A	109.0	C18—C19—C20	119.6 (5)
C5—C6—H6B	109.0	C18—C19—H19	120.2
C1—C6—H6B	109.0	C20—C19—H19	120.2
H6A—C6—H6B	107.8	C21—C20—C19	120.9 (6)
C8—C7—C9	110.6 (4)	C21—C20—H20	119.6
C8—C7—C1	113.2 (5)	C19—C20—H20	119.6
C9—C7—C1	111.5 (4)	C22—C21—C20	119.3 (5)
C8—C7—H7	107.0	C22—C21—H21	120.4
C9—C7—H7	107.0	C20—C21—H21	120.4
C1—C7—H7	107.0	C21—C22—C23	120.8 (5)
C7—C8—H8A	109.5	C21—C22—H22	119.6
C7—C8—H8B	109.5	C23—C22—H22	119.6
H8A—C8—H8B	109.5	C22—C23—C18	120.7 (5)
C7—C8—H8C	109.5	C22—C23—H23	119.6
H8A—C8—H8C	109.5	C18—C23—H23	119.6
H8B—C8—H8C	109.5		

### Hydrogen-bond geometry (Å, °)

**Table:**

*D*—H···*A*	*D*—H	H···*A*	*D*···*A*	*D*—H···*A*
C7—H7···O1	0.98	2.49	2.893 (5)	104
